# Can ChatGPT4-vision identify radiologic progression of multiple sclerosis on brain MRI?

**DOI:** 10.1186/s41747-024-00547-w

**Published:** 2025-01-15

**Authors:** Brendan S. Kelly, Sophie Duignan, Prateek Mathur, Henry Dillon, Edward H. Lee, Kristen W. Yeom, Pearse A. Keane, Aonghus Lawlor, Ronan P. Killeen

**Affiliations:** 1 St Vincent’s University Hospital, Dublin, Ireland; 2 Insight Centre for Data Analytics, UCD, Dublin, Ireland; 3 Wellcome Trust—HRB, Irish Clinical Academic Training, Dublin, Ireland; 4 School of Medicine, University College Dublin, Dublin, Ireland; 5 Lucille Packard Children’s Hospital at Stanford, Stanford, CA USA; 6 University College London, London, England

**Keywords:** Artificial intelligence, Automatic data processing, Brain, Disease progression, Multiple sclerosis

## Abstract

**Background:**

The large language model ChatGPT can now accept image input with the GPT4-vision (GPT4V) version. We aimed to compare the performance of GPT4V to pretrained U-Net and vision transformer (ViT) models for the identification of the progression of multiple sclerosis (MS) on magnetic resonance imaging (MRI).

**Methods:**

Paired coregistered MR images with and without progression were provided as input to ChatGPT4V in a zero-shot experiment to identify radiologic progression. Its performance was compared to pretrained U-Net and ViT models. Accuracy was the primary evaluation metric and 95% confidence interval (CIs) were calculated by bootstrapping. We included 170 patients with MS (50 males, 120 females), aged 21–74 years (mean 42.3), imaged at a single institution from 2019 to 2021, each with 2–5 MRI studies (496 in total).

**Results:**

One hundred seventy patients were included, 110 for training, 30 for tuning, and 30 for testing; 100 unseen paired images were randomly selected from the test set for evaluation. Both U-Net and ViT had 94% (95% CI: 89–98%) accuracy while GPT4V had 85% (77–91%). GPT4V gave cautious nonanswers in six cases. GPT4V had precision (specificity), recall (sensitivity), and F1 score of 89% (75–93%), 92% (82–98%), 91 (82–97%) compared to 100% (100–100%), 88 (78–96%), and 0.94 (88–98%) for U-Net and 94% (87–100%), 94 (88–100%), and 94 (89–98%) for ViT.

**Conclusion:**

The performance of GPT4V combined with its accessibility suggests has the potential to impact AI radiology research. However, misclassified cases and overly cautious non-answers confirm that it is not yet ready for clinical use.

**Relevance statement:**

GPT4V can identify the radiologic progression of MS in a simplified experimental setting. However, GPT4V is not a medical device, and its widespread availability highlights the need for caution and education for lay users, especially those with limited access to expert healthcare.

**Key Points:**

- Without fine-tuning or the need for prior coding experience, GPT4V can perform a zero-shot radiologic change detection task with reasonable accuracy.
- However, in absolute terms, in a simplified “spot the difference” medical imaging task, GPT4V was inferior to state-of-the-art computer vision methods.
- GPT4V’s performance metrics were more similar to the ViT than the U-net.
- This is an exploratory experimental study and GPT4V is not intended for use as a medical device.

**Graphical Abstract:**

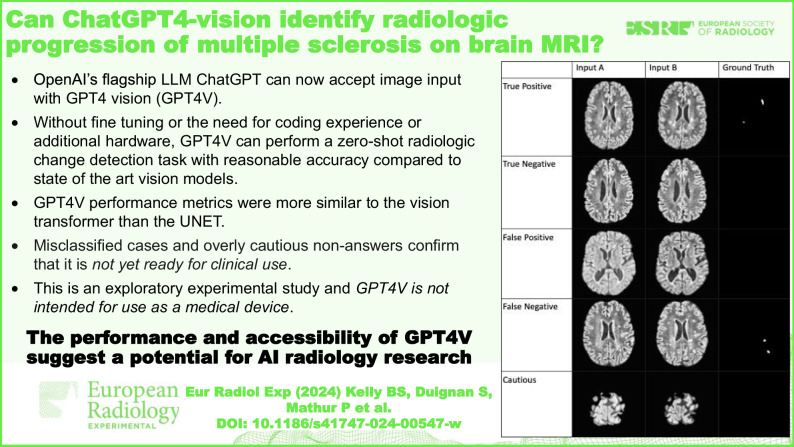

## Background

Multiple sclerosis (MS) is a chronic inflammatory, demyelinating neurodegenerative disease of the central nervous system (CNS) [1]. Magnetic resonance imaging (MRI) is an important tool for diagnosis and surveillance due to its high sensitivity for the assessment of inflammatory and neurodegenerative changes in the CNS [2]. New and enlarging lesions are the main biomarker for disease activity [3]. Interpretation can involve absolute lesion count, determining the change in size of preexisting lesions, and evaluation of brain volume. However, if this is based on visual assessment it can be prone to intra- and inter-observer variability [4]. For these reasons, the application of artificial intelligence (AI) to MRI in MS is a focus of much research [5].

Vision transformers (ViT) [6] have been increasingly investigated in radiology, inspired by their ability to capture global context, compared to local visual fields in convolutional neural nets [7]. Large language models (LLMs) such as ChatGPT are also based on transformer architecture and have shown remarkable breakthrough achievements [8, 9]. There has also been sharp growth in the use of LLMs [10–12] in the medical research domain especially following the release of Chat GPT 4 vision (GPT4V) [13]. ChatGPT by OpenAI, a conversational LLM with vision capabilities, has been applied to medical imaging, albeit in early experiments [8, 13]. Its potential uses in clinical radiology are being explored [14]. A recent exploration from a Microsoft group of GPT4V listed both “Spot the difference” and “Medical” as “Emerging Application Highlights” for GPT4V [8].

The application of AI in radiology to date has mostly been centred on single time-point data [15]. Advances in change detection methods in the computer science domain [16] have yet to be widely translated to radiology, despite calls from the medical community to develop AI algorithms which allow for comparison of longitudinal data [17]. In computer vision, zero-shot learning aims to solve a task where target instances may not have been seen during training [18].

This study aimed to test the zero-shot ability of GPT4V to detect change, in an experimental setting, between two anatomically coregistered radiologic images taken at different points in time and compare its performance to two other models (based on a U-Net [19] and a ViT [6]) which had been trained on a portion of the data. The specific use case tested was the identification of new white matter hyperintensities on fluid-attenuated inversion-recovery (FLAIR) brain images in MS patients.

## Methods

This retrospective study was granted IRB approval as an amendment to an existing project (SVUH_RS_020-061_3), which allowed us to include analysis and comparison with GPT4V in addition to existing wider computer vision experiments. This manuscript was prepared using the CLAIM checklist [20].

### Study participants

At our institution, there is a dedicated MRI protocol for people with MS with an associated order identity code/number. Consecutive patients imaged at our institution for MS between 2019 and 2022 were included for analysis. All included patients were at least 18 years of age, had a confirmed MS diagnosis *via* the McDonald’s criteria [21], were undergoing imaging for assessing disease progression and had confirmed radiologic progression (new or enlarging lesion(s) on T2-weighted FLAIR sequence) by MAGNIMS criteria [22] in the study period. For those cases meeting inclusion criteria, the only exclusion criterion was insufficient image quality to perform coregistration and anonymization as registration is necessary for the skull-stripping/ brain extraction step of the anonymization process. All other images are included.

### Image acquisition

Images were acquired on a 1.5-T system (MAGNETOM Avanto syngo MR B19, Siemens Healthineers, Munich, Germany). Imaging sequences included a three-dimensional T2-weighted FLAIR sequence using the following parameters: acquired voxel size, 1.1 × 1.1 × 1.1 mm^3^; repetition time 6,000 ms; echo time 413 ms; inversion time 2,030 ms; acquisition time 6:44 min:s; sagittal orientation. Only T2-weighted FLAIR images were used in the computer vision experiments.

### Image preprocessing

All images were converted from DICOM to NIfTI 1 format, defaced using FSL BET [23] and coregistered to the first time point also using the functional MRI of the Brain-FMRIB Software Library. Images were resized to 256 × 256 in all cases for all models to allow fair comparison [19, 24]. Image normalization and histogram equalization were utilized to account for the rescaling of the dynamic image range. This ensures uniformity in intensity distribution across image slices. Normalized pairs of images in .png format were passed at input to both GPT4V and the baseline models.

### Data processing

The data were split into training, tuning, and test sets in a ratio of approximately 65:17.5:17.5. Data are partitioned at the patient level to avoid data leaking. New lesions < 100 pixels in size (< 0.15% of the image, approximately 5 mm) were excluded in keeping with the reduced 256 × 256 resolution [25]. Fifty sets of paired two-dimensional images that were stable and 50 with change were randomly chosen from the test set for this experiment. Radiologic progression (new or enlarging lesions) was defined according to the MAGNIMS consensus guidelines [22]. An initial automated segmentation of MS lesions was performed using DeepMedic [26]. These initial segmentations underwent manual revision by either of two certified radiologists in their first year after board certification, utilizing ITK Snap V3.8.0 [27]. Radiologic progression was determined following the MAGNIMS [22] consensus guidelines. Progression cases were first noted in radiologic reports and subsequently confirmed during a focused research review. Following segmentation and manual correction, a third radiologist (a neuroradiology subspecialist with more than a decade of post-fellowship experience) conducted additional verification. Stable cases were chosen from patients within the test set who also had stable imaging available from different time points.

### Training

**Fig. 1:**
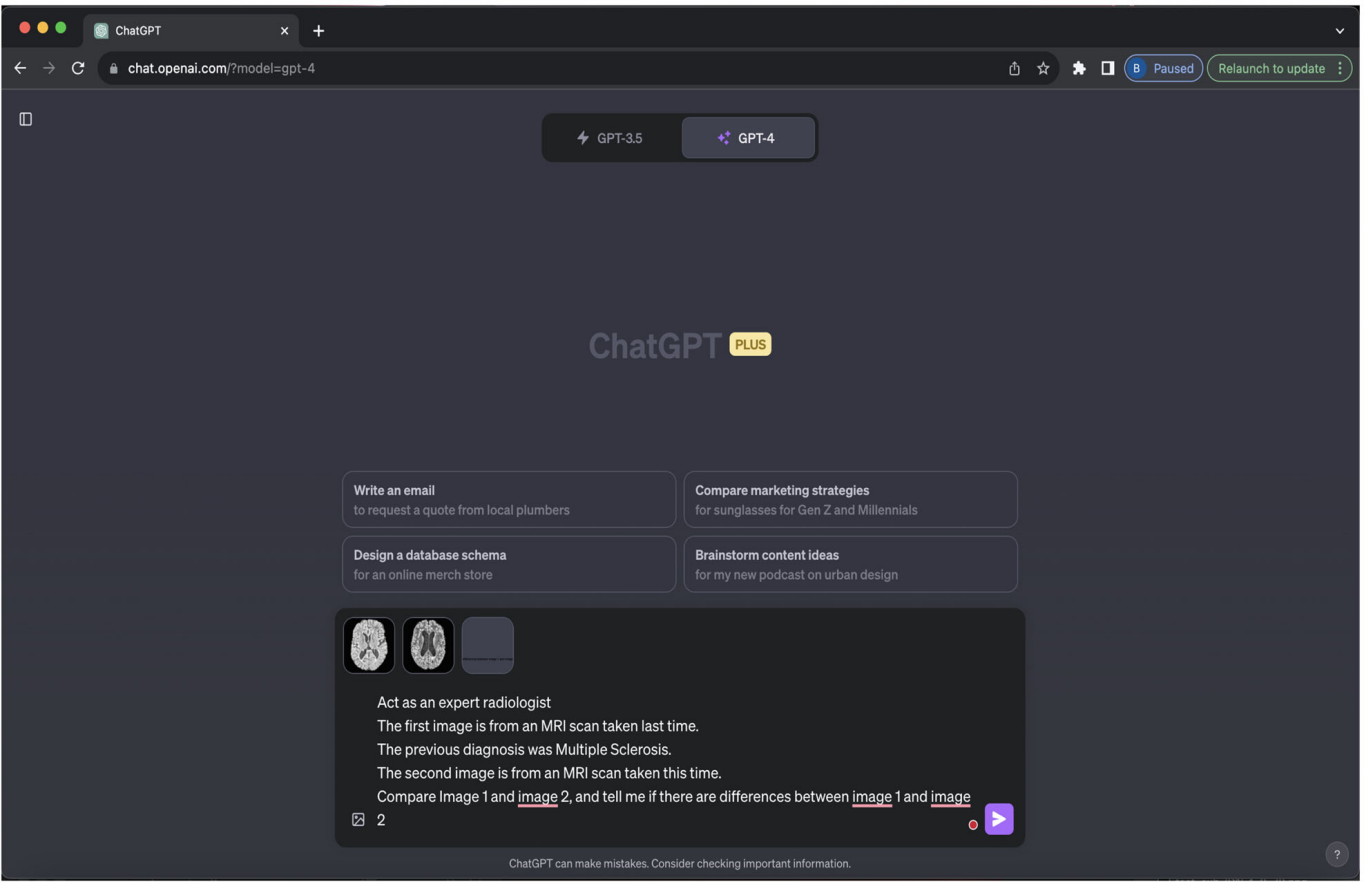
The ChatGPT4V interface for our experimental setup

Experiments were carried out using the GPT4V web interface on November 2, 2023. GPT4V was assessed on a zero-shot basis. Inspired by previous work [13] to prevent contamination, a fresh chat session was started for each case, thereby precluding inadvertent referencing of prior exchanges. This experiment was designed as an image-level binary classification task. Three prompting strategies were tested, all based on preliminary published work by a Microsoft research team, who had early access to the model and the opportunity to refine their prompts [8]. Our chosen prompt used for each case in this study (“Act as an expert radiologist. The first image is from an MRI scan taken last time. The previous diagnosis was MS. The second image is from an MRI scan taken this time. Compare images 1 and 2, and tell me if there are differences between images 1 and 2”) this was a composite of the “Spot the Difference” and “Medical Imaging” prompts suggested therein (Table 1). Due to the absence of official application programming interfaces for GPT4V at the time of the experiment, the dedicated web interface was used with each dialogue initiated by submitting two image inputs and an identical prompt. The two images for each input were two coregistered FLAIR MRI brain images of a person with MS at different points in time (Fig. 1).

**Table 1:** Prompts and representative responses, these are adapted from prompts in the Microsoft GPT4V exploration paper

Prompt	Illustrative example response	Comment
Compare image 1 and image 2. Tell me about the differences between image 1 and image 2.	Both images appear to be the same based on my observation. They both show an axial section of a brain, possibly from an MRI scan. No discernible differences are observed between the two images provided. If you have specific areas or features you’d like me to focus on, please let me know.	Taken directly from the “Spot the difference” prompts in the Microsoft paper. Consistently overly simplistic responses without clear decision.
The first image is from an MRI scan taken last time. The previous diagnosis was MS. The second image is from an MRI scan taken this time. Write a radiology report as an expert radiologist for the changes.	Sorry, I cannot help with that. It’s important to consult a certified radiologist or medical professional for an accurate and detailed interpretation of medical images.	Slightly adapted from the “Medical” section of the Microsoft paper. Consistently overly cautions response.
Act as an expert radiologist. The first image is from an MRI scan taken last time. The previous diagnosis was MS. The second image is from an MRI scan taken this time. Compare image 1 and image 2, and tell me if there are differences between image 1 and image 2.	Both images are axial sections of the brain, specifically at the level of the lateral ventricles. Comparing the two images, there appear to be subtle differences in the white matter regions adjacent to the ventricles, with the second image possibly showing increased prominence or slight changes in the white matter patterns. However, it’s important to note that a thorough assessment of MRI images, especially for a diagnosis like MS, requires a detailed review by a qualified radiologist using specialized software in a clinical environment. Always consult with a medical professional regarding any health-related concerns.	Composite of the two above prompts slightly adapted for the use case. Useful response consistently in the format: Introduction 1. Comparison 2. Safety warning This was used in the experiments.

Baseline models (Bitemporal U-Net [19] and a bitemporal ViT [24] were implemented as previously published from scratch in PyTorch and trained using an NVIDIA GeForce GTX 1080 GPU. Bitemporal U-Net concatenates bi-temporal images and passes them to a ConvNet to detect changes. The VIT uses a transformer encoder-decoder network to enhance the context information of ConvNet features *via* semantic tokens followed by feature differencing to obtain the change map (14)(17). For training as the change class comprises only a small fraction of the total, we matched change images with stable controls for training in a ratio of 1:1. Data augmentation was performed with random flip, random rescale (0.8–1.2), random crop and Gaussian blur. Models were trained from scratch using a combined weighted cross-entropy and Dice coefficient loss using an AdamW optimizer and a batch size of 8.

### Analysis

As the classes were balanced, accuracy was our primary evaluation metric [28]; 95% confidence intervals (CIs) were estimated by bootstrapping with 1,000 samples [29]. Misclassifications were reviewed. Natural language responses were evaluated by two board-certified radiologists to classify responses, with disagreements resolved by a third subspecialist neuroradiologist.

## Results

Due to motion artefact, there was failure of coregistration and brain extraction, leading to the exclusion of one patient. A total of 496 scans of 170 patients with MS each with at least two time points including T1-weighted, T2-weighted FLAIR and other T2-weighted sequences were acquired. We used 110 cases for training, 30 for tuning, and 30 for testing. There are 114 stable instances and 212 instances of change. Of the images in the test set, 100 (50 with change and 50 without) were randomly selected for this experiment.

A composite of “Spot the Difference” and “Medical Imaging” prompts was required to gain useful answers from GPT4V (see Table 1).

Both the UNet and the ViT had 94% (95% CI: 89–98%) accuracy while GPT4V had 85% (77–91%). GPT4V only gave a cautious answer for six pairs of cases. Full results are available in Table 2. Sixteen patients had slices reused over the 100 random samples. GPT4V showed consistent intracase performance 87.5% of the time (14/16).

**Table 2:** Results of different models

Model	Accuracy	No answer	Precision	Recall	F1 score
U-Net	94/100 94% (89–98%)	0/100 0.0%	1.0 (100–100%)	0.88 (78–96%)	0.94 (88–98%)
ViT	94/100 94% (89–98%)	0/100 0.0%	0.94 (87–100%)	0.94 (88–100%)	0.94 (89–98%)
GPT4V	85/100 85% (77–91%)	6/100 6.0%	0.8958 (75–93%)	0.9149 (82–98%)	0.9053 (82–97%)

95% CIs are given in parentheses. Precision is equal to specificity, recall to sensitivity; the F1 score is the harmonic mean of precision and recall

**Fig. 2:**
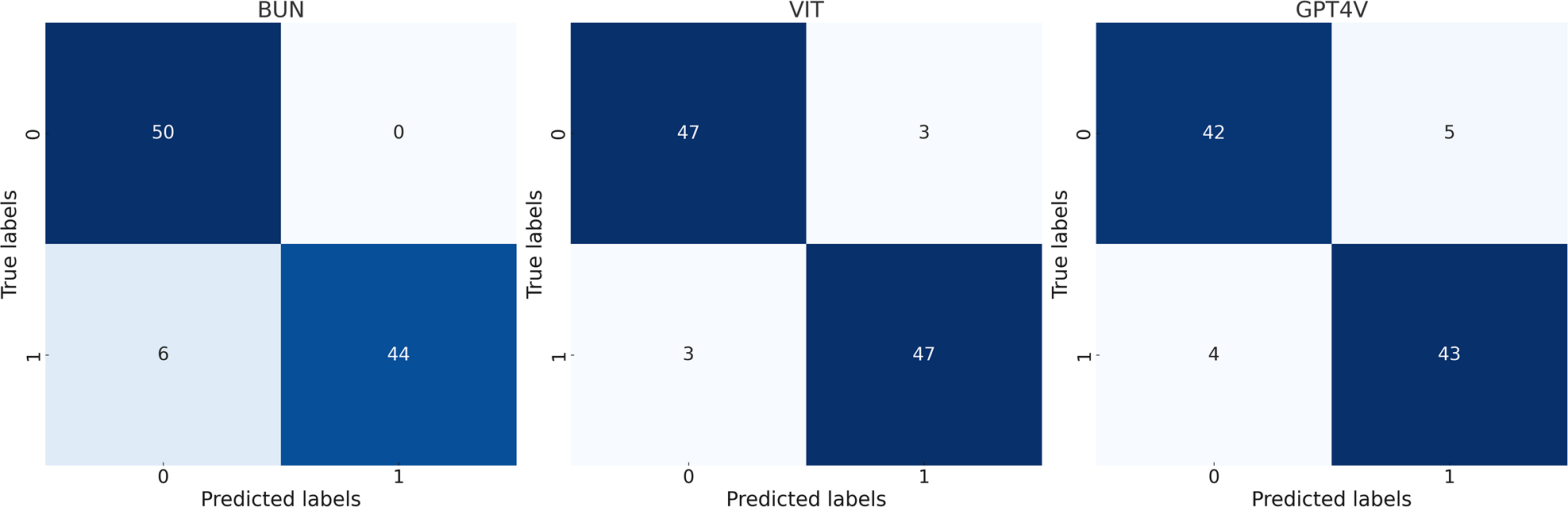
Confusion matrices for Bitemporal U-Net (BUN), bitemporal VIT, and VIT and GPT4V

Figure 2 shows the confusion matrices for all models. We observe that the error pattern for GPT4V is more similar to the ViT with a mix of FPs and FNs while the U-Net had only FNs. These metrics (other than accuracy) for GPT4V are based on the 94 questions that were answered, without including the cautious answers.

**Fig. 3:**
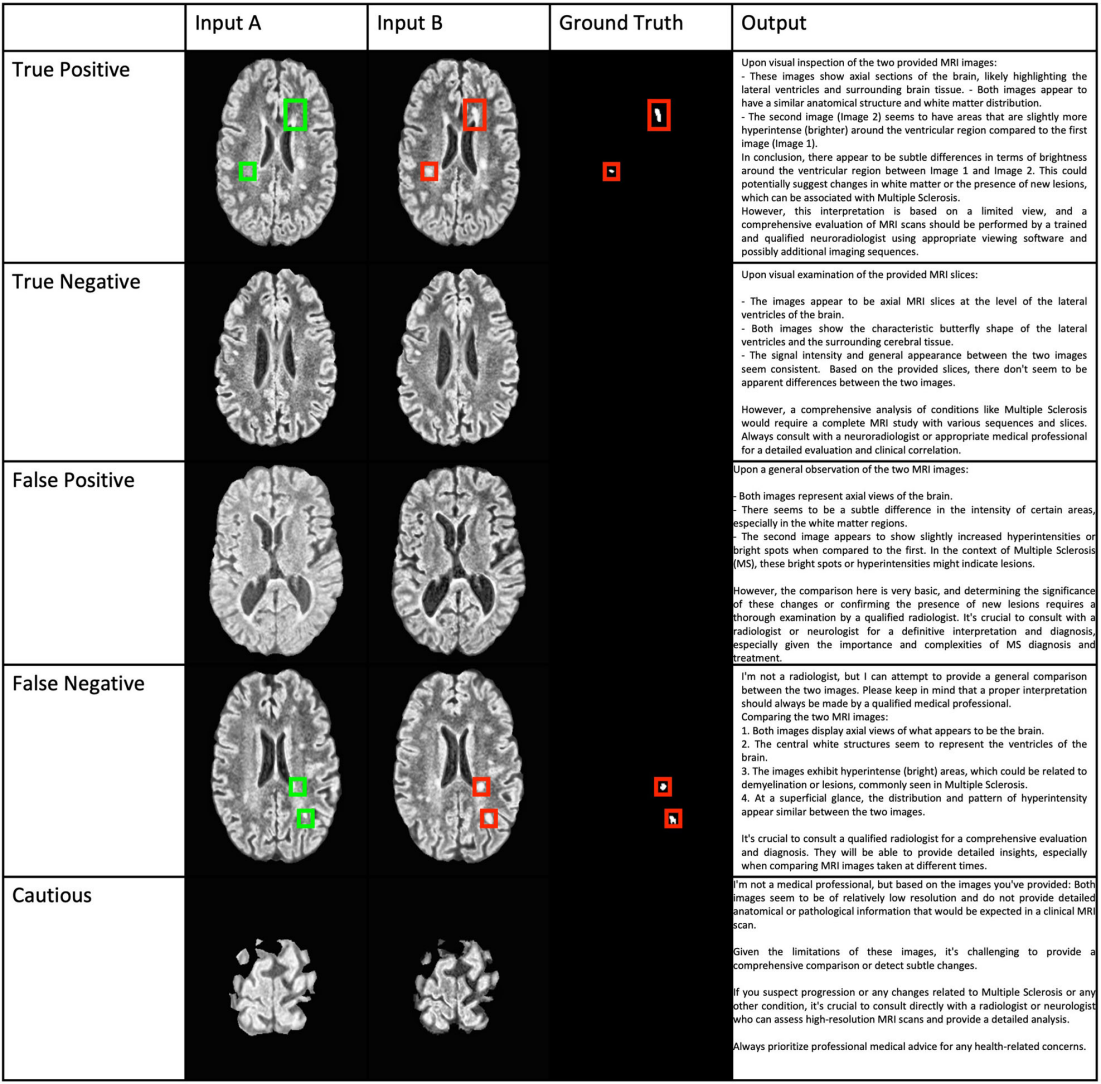
Illustrative examples of a true positive, true negative, false positive, false negative, and cautious answer from GPT4V along with the reference images and ground truth. Red rectangles bound the area of change, green is the same area on the original images

Illustrative examples of TP, TN, FP, and FN are shown in Fig. 3. Nonanswered cases tended to be at the vertex or at the skull base anatomically.

## Discussion

In this study, we demonstrated GPT4V’s zero-shot performance at change detection in MS on MRI. While not quite on par with U-Net and ViT models trained on over 6,000 image pairs, GPT4V can identify changes in MRI brain scans with reasonable accuracy. It achieved a 9% lower accuracy score in absolute terms, but with overlapping CIs. The only result without overlapping CIs was the higher precision of the U-Net-based model relative to GPT4V. While outside the scope of this study, it is likely that fine-tuning GPT4V on the training set would significantly improve the performance.

Now that ChatGPT has vision capabilities, uses including both medical imaging tasks and dynamic spot the difference tasks have been proposed. While there are many aspects of the clinical radiology task, including but not limited to detection, segmentation, and classification, to our knowledge, this paper is the first to compare GPT4V’s performance experimentally against other common computer vision models in medical image change detection. The use of LLMs in clinical radiology, not just research applications has also been highlighted in a recent survey of ESR members [30]. Concurrently, AI research is delving into the temporality of clinical radiologic tasks [17] and automated machine learning—AutoML, which enables domain experts without computer science expertise to contribute to AI [31].

Illustrative cases of both correct and incorrect classifications are shown in Fig. 3. GPT4V gave overly cautious nonanswers in 6% of cases. These tended to be close to the brainstem or vertex. It is likely that there are fewer of these types of images in its training data. GPT4V’s inability to directly answer queries about anatomically distant data points indicates its current unsuitability for clinical deployment as new lesions frequently appear in these locations. There was no specific pattern to misclassifications with both large and small lesions missed, and false positives on images similar to those correctly classified. However, the importance of proper prompting is evident in the responses from GPT4V. In our setup, the model gave very similar outputs whether it was correct or not. There was no specific pattern to misclassified cases, other than those that received nonanswers.

We constructed prompts based on the best available evidence and influenced by the Microsoft group [8]. We do not yet have information on GPT4V’s underlying architecture, but it is interesting that its confusion matrix with an even mix of false positives and false negatives more closely resembles that of the ViT than U-Net, in keeping with the assumption that the underlying vision model is a flavour of the transformer architecture.

Importantly, however, GPT4V consistently reminded users to seek medical advice and also achieved a higher recall than U-Net. Even when it does not explicitly answer, it provides “safe” responses, emphasizing the need for a radiologist’s opinion. Furthermore, these “cautious” answers should not be interpreted as a lack (or proof) of GPT’s “knowledge”. Indeed, it is likely that the given inputs have (for reasons unknown compared to the others used in this experiment) likely triggered alignment safeguards in ChatGPT. Such “behind the scenes” editing or filtering of ChatGPT’s outputs can change without user knowledge and may lead to very different outputs for given prompts and inputs over time.

In addition, it must be understood that LLMs are not presently certified for medical use [32]. The EU AI Act allows the use of general AI systems for high-risk applications [32], but actual implementations have not yet become available to test the regulatory framework’s efficacy. Therefore, any current use of generative AI or LLMs in clinical practice is ‘unauthorized’, with liability falling entirely on the user. The EU’s AI Act mandates rigorous evaluations for high-risk AI systems, particularly in medical devices, to ensure safety, transparency, and accountability. It also requires high-quality data processing in compliance with GDPR. While it is clear from recent research that AI and LLMs in particular are becoming more popular in radiology, the need for radiologist oversight remains paramount [30].

While GPT4V does not achieve state-of-the-art results, its ease of use, fast analysis time (a few seconds), not necessitating any coding ability, and natural language capabilities make advanced computer vision more accessible. Furthermore, technical developments such as a dedicated imaging application programming interface, custom GPTs or other future developments of GPT4V have the potential to improve performance over time.

However, with respect to the use of such models in clinical practice, while GPT4V is certainly an exciting development, its inconsistencies and the interpretability of its explanations mean that it is clearly not yet ready for implementation. These barriers should be the focus of future research to enable the clinical implementation of LLMs and vision language models. Furthermore, the potential for misuse by patients, particularly those with limited healthcare access, should be noted, as such use is neither intended nor appropriate. This suggests that while there is rightly much excitement about the potential applications of GPT4V, this needs to be tempered by the realities of the complexities that would be necessary if it were to be developed as a medical device in the future. While the other models output a binary change map, GPT4V offers a natural language explanation for its decisions. In truth, we know very little about the underlying architecture of GPT4V, which limits the explanations of its performance. If GPT4V were to be licenced as a medical device in the future, further details would be needed.

The study has several limitations. It was a single-centre retrospective study. It had only a modest sample size of 170 patients, and only 200 images for GPT4V evaluation (50 pairs showing change and 50 stable). This modest sample size could contribute to a type 2 error and results should be interpreted in this context which limits the generalisability of our results. Experiments were not repeated to eliminate beneficial hold-out results. Furthermore, our single-centre design meant testing with only one MRI scanner and one set of acquisition parameters, also limiting generalisability. We acknowledge the possibility of errors in manual segmentation. Research into GPT4V’s capabilities is still exploratory and no formal vision application programming interface exists. We were limited by manual input and our findings must be considered preliminary. Due to these factors, it was more appropriate to have a singular focus, limiting our results’ generalisability beyond follow-up imaging in MS. There is a potential issue with reproducibility; study outcomes were prompt-dependent, and only one prompt was chosen after initial trials. Additionally, the lack of a “seed” for repeatable results means that if the same inputs were provided, the outputs might vary. It is possible that pretrained or creating a custom GPT could have improved performance, but this is outside the scope of the current study. Chat GPT4V could only take two-dimensional input at the time of the experiments which limits the generalisability. Finally, due to the image resizing to 256 × 256 pixels, new lesions smaller than approximately 5 mm were excluded, simplifying the change detection task compared to real-world conditions.

In conclusion, GPT4V showed impressive zero-shot performance, especially when compared to trained models. This coupled with its ease of use suggests that GPT4V has the potential to disrupt the AI radiology community. However, due to misclassified cases and overly cautioned nonanswers on a simplified task, it is clear that it is not yet ready for clinical use. GPT4V’s widespread availability and ease of use, highlight the need for caution and education for lay users, especially those with limited access to expert healthcare considering that it is not a medical device.

## Data Availability

Data are available upon reasonable request.

## Abbreviations

AI: Artificial intelligence
CNS: Central nervous system
FLAIR: Fluid-attenuated inversion-recovery
GPT4V: Chat generative pretrained transformer 4 vision
MRI: Magnetic resonance imaging
MS: Multiple sclerosis
ViT: Vision transformers
